# Relapsing-remitting multiple sclerosis patients display an altered lipoprotein profile with dysfunctional HDL

**DOI:** 10.1038/srep43410

**Published:** 2017-02-23

**Authors:** Winde Jorissen, Elien Wouters, Jeroen F. Bogie, Tim Vanmierlo, Jean-Paul Noben, Denis Sviridov, Niels Hellings, Veerle Somers, Roland Valcke, Bart Vanwijmeersch, Piet Stinissen, Monique T. Mulder, Alan T. Remaley, Jerome J. A. Hendriks

**Affiliations:** 1 Hasselt University, Dept. of Immunology and Biochemistry, Biomed, Diepenbeek, Belgium; 2 NIH, Dept. of Laboratory Medicine, Clinical Center, Bethesda, United States; 3 Hasselt University, Faculty of Sciences, Molecular and Physical Plant Physiology, Diepenbeek, Belgium; 4 Revalidation and MS Center, Overpelt, Belgium; 5 Erasmus MC, Dept. of Vasc. and Met. diseases, Rotterdam, the Netherlands

## Abstract

Lipoproteins modulate innate and adaptive immune responses. In the chronic inflammatory disease multiple sclerosis (MS), reports on lipoprotein level alterations are inconsistent and it is unclear whether lipoprotein function is affected. Using nuclear magnetic resonance (NMR) spectroscopy, we analysed the lipoprotein profile of relapsing-remitting (RR) MS patients, progressive MS patients and healthy controls (HC). We observed smaller LDL in RRMS patients compared to healthy controls and to progressive MS patients. Furthermore, low-BMI (BMI ≤ 23 kg/m^2^) RRMS patients show increased levels of small HDL (sHDL), accompanied by larger, triglyceride (TG)-rich VLDL, and a higher lipoprotein insulin resistance (LP-IR) index. These alterations coincide with a reduced serum capacity to accept cholesterol via ATP-binding cassette (ABC) transporter G1, an impaired ability of HDL_3_ to suppress inflammatory activity of human monocytes, and modifications of HDL_3_’s main protein component ApoA-I. In summary, lipoprotein levels and function are altered in RRMS patients, especially in low-BMI patients, which may contribute to disease progression in these patients.

Lipoproteins are crucial mediators of cholesterol transport and play an important role in the regulation of inflammatory responses. High density lipoprotein (HDL) has anti-atherogenic properties that are primarily attributed to its key role in reverse cholesterol transport. Furthermore, HDL has anti-inflammatory effects on monocytes and endothelial cells, has anti-oxidant properties, and HDL’s main associated protein, ApoA-I, reduces inflammation in the central nervous system (CNS) by preventing contact between T cells and macrophages^1^,^2^,^3^,^4^. HDL consists of heterogeneous subclasses which can be identified based on their density, charge, size, and protein composition^5^. Importantly, changes in HDL subclass distribution go together with alterations in the levels of other plasma lipoproteins^6^,^7^,^8^,^9^, and are often associated with HDL dysfunction as is observed in chronic inflammatory diseases like type 2 diabetes (T2D) and atherosclerosis^10^,^11^,^12^,^13^,^14^,^15^,^16^,^17^,^18^,^19^,^20^,^21^,^22^,^23^,^24^,^25^.

Multiple sclerosis (MS) is an autoimmune disease, characterized by chronic inflammation and demyelination in the central nervous system (CNS). Relapsing-remitting MS (RRMS) is the most frequent (80–90%) occurring type of MS, and is characterized by unpredictable periods of inflammatory relapse and remission phases. In most RRMS patients, the disease gradually progresses with an increased number of relapses (i.e. Progressive Relapsing MS (PRMS)), towards a progressive disease course characterized by more prominent role for neurodegeneration compared to inflammation (i.e. secondary progressive MS (SPMS))^26^. The onset and progression of multiple sclerosis (MS) is presumed to be driven by an autoreactive immune response. HDL may interfere with these processes by multiple mechanisms such as its ability to modulate monocyte and T cell responses^3^,^27^,^28^,^29^,^30^.

Despite the chronic inflammatory character of MS, it is poorly understood if and how lipoprotein levels, subclasses, and function are altered in MS patients, and whether such changes influence disease progression. Notably, Penesova and colleagues recently described decreased insulin sensitivity and postprandial hyperinsulinemia in MS patients^31^. Reduced insulin sensitivity is typically associated with lipoprotein abnormalities^32^. Interestingly, a positive association between patient disability and plasma LDL, ApoB, and total cholesterol levels is observed in MS patients^33^,^34^,^35^. In addition, higher serum HDL was found to be associated with lower levels of blood-brain-barrier injury and decreased cell extravasation into the CSF^30^. Reports on HDL levels in MS patients are however inconsistent. Whereas some studies report an increase^36^,^37^, others suggest a decrease^38^, or show no change in HDL levels^39^. These contradictory findings may be partially explained by the lack of distinction between different HDL subclasses. Importantly, increased levels of oxidized LDL in the plasma and CNS^40^,^41^ and higher serum levels of auto-antibodies against oxidized LDL^39^ are observed in MS patients. In line with this, the loss of HDL anti-oxidant function in MS patients is suggested^41^,^42^, indicating that lipoprotein function may also be affected.

In this study, nuclear magnetic resonance (NMR) spectroscopy was used to determine the lipoprotein profile of relapsing-remitting MS (RRMS) patients, progressive MS patients and healthy controls (HC). Furthermore, HDL function was assessed and HDL’s main protein component, ApoA-I, was analyzed using liquid chromatography–mass spectrometry (LC-MS/MS). We show an altered lipoprotein profile in RRMS patients, especially pronounced in low-BMI RRMS patients, with dysfunctional HDL that is modified at its ApoA-I tyrosine and tryptophan residues.

## Results

### RRMS patients have smaller LDL particles

The different lipoproteins and their subclasses, as well as a lipoprotein-based insulin resistance index (LP-IR) were determined in RRMS patients, progressive MS patients, and healthy controls by NMR. Table 1 provides an overview of characteristics of the study population and of their lipid and lipoprotein profile. RRMS patients show smaller LDL and in line with this a lower level of large LDL particles compared to controls and progressive MS patients. No other significant differences between the groups were found for the measured lipoprotein subclasses, LP-IR index, BMI, or male-to-female ratios. Progressive MS patients were significantly older (P < 0.01) than HC and RRMS patients. Therefore, age was added as a possible covariate to the regression models in further analyses.

### RRMS patients with a low BMI have smaller HDL particles

**Figure 1:**
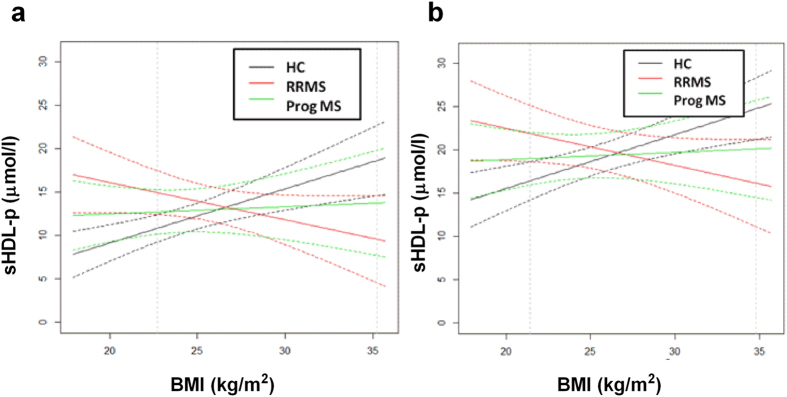
RRMS patients display an inverse relationship between BMI and sHDL-p. The regression line describing the relationship between sHDL and BMI, distinguishing between groups (i.e. HC (n = 89, black), RRMS (n = 35, red), and Prog MS (n = 25, green)), for females (**a**) and males (**b**) is shown. The dotted vertical line shows the threshold for BMI for which RRMS patients and HC differ significantly in their amount of sHDL-p. - p = particle count.

To correct the measured lipoprotein parameters for the difference in age and to determine possible other confounding effects of the independent covariates “gender”, “BMI”, and “group”, and possible interactions between these covariates, we performed univariate multiple linear regression analyses for each measured lipoprotein subclass. The lipoprotein parameters for which a significant contribution of covariates was found, are shown in Table 2. Based on the fitted models, the mean size of LDL (LDL-z, nm) is smaller in RRMS patients compared to healthy controls and progressive MS patients of the same gender. Furthermore, RRMS patients have a lower amount of large LDL particles (Large LDL-p, nmol/l) compared to controls and progressive MS patients of the same age and gender. In addition, sHDL particle count (sHDL-p, μmol/L) is increased in the plasma of RRMS patients within a distinct BMI range compared to controls with the same BMI. More specific, RRMS patients with a low BMI ( ≤ 23 kg/m^2^) displayed an increase in sHDL compared to low-BMI controls of the same gender. The BMI threshold for differences in sHDL was determined based on the confidence bands of the regression lines describing the relationship between sHDL and BMI, distinguishing between groups (Fig. 1), and was similar for male and female subjects. Interestingly, these regression lines show a negative relation between sHDL-p and BMI for RRMS patients, whereas this relation is positive for HC, and neutral for progressive MS patients. Within each group (e.g. RRMS, progressive MS and controls), lipoprotein alterations were more pronounced in male than in female subjects. Altogether, our data show that LDL particle distribution of RRMS patients is shifted towards smaller LDL particles as they show a decrease in large LDL, together with a decrease in LDL size. In addition, low-BMI RRMS patients display a shift towards increased levels of sHDL.

### RRMS patients with a low BMI display an insulin resistant-like lipoprotein profile

An increase in the amount of sHDL is often accompanied by other changes in the lipoprotein profile, such as observed in patients with T2D^43^,^44^. For this reason, we performed univariate multiple linear regression analyses for all of the lipoprotein parameters measured with NMR for low-BMI controls (n = 29), RRMS patients (n = 10) and progressive MS (n = 11) patients. In addition to the above mentioned increase in sHDL, RRMS patients with a low BMI showed a higher LP-IR, larger VLDL particles (nm), and an increased amount of TG (mg/dl) and VLDL-TG (mg/dl) compared to controls and to progressive MS patients (Table 3). No differences were found in HDL cholesterol (HDL-c) or LDL-c levels. Interestingly, a positive correlation between the amount of sHDL-p and sLDL-p levels in RRMS patients with a low BMI (r = 0.5, P = 0.05) was observed, and the LP-IR of these patients correlated negatively with HDL-c (r = −0.6, P = 0.05), and positively with concentrations of TG (r = 0.7, P = 0.01) and VLDL-TG (r = 0.7, P = 0.01) (data not shown), hereby highlighting the interdependency of these lipoprotein parameters. Altogether, these data show that lipoprotein levels are especially altered in RRMS patients with a low BMI.

### RRMS patients show normal LCAT mass

**Figure 2:**
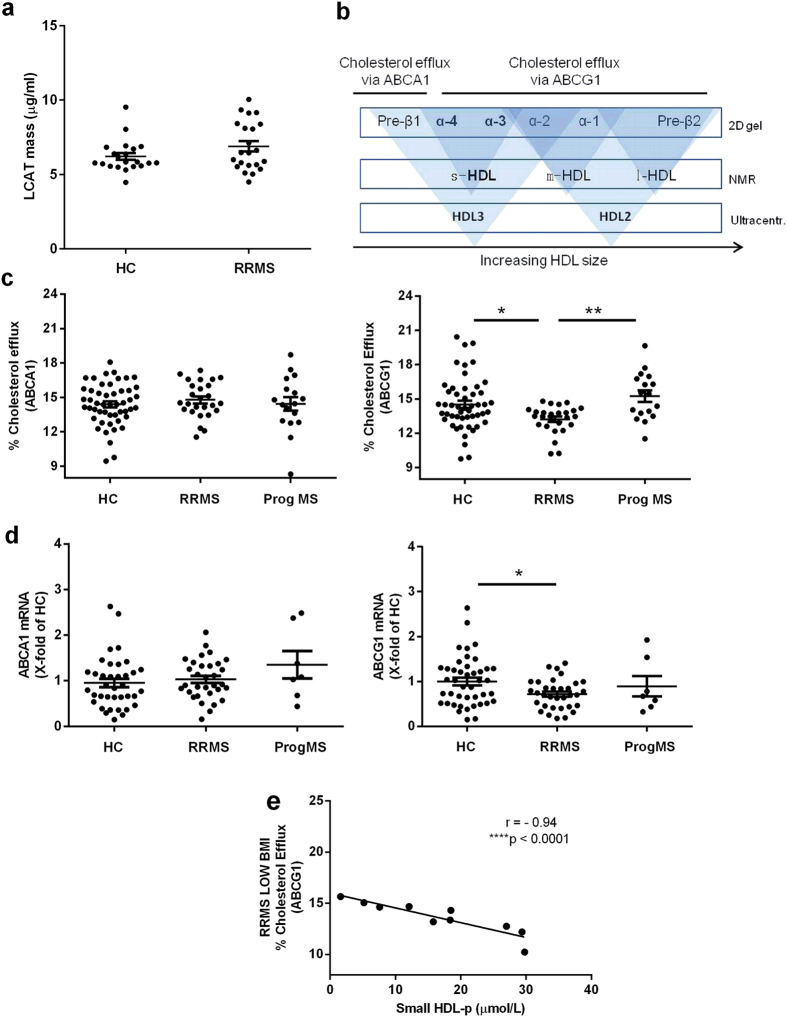
RRMS patients have reduced serum cholesterol efflux capacity via ABCG1 and reduced monocyte ABCG1 mRNA expression compared to healthy controls. (**a**) LCAT mass (μg/ml) was measured in freshly thawed plasma samples of HC (n = 21) and RRMS (n = 22) patients using ELISA. (**b**) Schematic representation of sHDL measured by NMR, based on 2D gelelectrophoresis, and ultracentrifugal separation. (**c**) Capacity of serum from controls (n = 50), RRMS (n = 26), and progressive MS (n = 17) patients to accept cholesterol via the ABCA1 (left) or ABCG1 (right) transporters from ^3^H-cholesterol loaded BHK cells transfected with human ABCA1 or ABCG1 respectively. (**d**) Basal ABCA1 (left) and ABCG1 (right) mRNA expression in monocytes of HC (n = 43), RRMS (n = 33), and progressive MS (n = 7) patients measured using quantitative PCR (qPCR). Results are expressed as fold change of healthy controls. (**e**), Pearson correlation coefficient for percentage serum cholesterol efflux capacity via the ABCG1 transporter versus sHDL (μmol/L) for RRMS patients with a low BMI (n = 10). *P < 0.05, ****P < 0.0001.

Lecithin - cholesterol acyltransferase (LCAT) mediates the esterification rate of free cholesterol onto lipid poor HDL particles, hereby increasing the size of HDL particles. LCAT mass is highly correlated with its enzyme activity and with its molar cholesterol esterification rate^45^. RRMS patients did not show alterations in LCAT mass compared to healthy controls (Fig. 2a), indicating that the observed increase in sHDL in RRMS patients cannot be explained by alterations in LCAT mass. Furthermore, age, gender, and BMI had no confounding effect on LCAT mass.

### Serum of RRMS patients has a reduced capacity to accept cholesterol via the ABCG1 transporter

Cellular reverse cholesterol transport determines the inflammatory phenotype of monocytes. sHDL measured by NMR in our study are predominantly alpha 3 (α−3) and alpha 4 (α−4) HDL particles that efflux cholesterol via the ATP-binding cassette G1 (ABCG1) transporter (Fig. 2b). To determine whether changes in the quantity of sHDL in RRMS patients with a low BMI are associated with changes in HDL function, we made use of a physiologically relevant assay to analyze the capacity of serum from RRMS patients to accept cholesterol from ^3^H-cholesterol labeled BHK cells transfected with human ABCA1 or ABCG1. Our data show that the capacity of serum lipoproteins to accept cholesterol via the ABCA1 transporter is similar between healthy controls, RRMS, and progressive MS patients (Fig. 2c). However, serum cholesterol efflux capacity via the ABCG1 transporter was reduced in RRMS patients compared to controls and progressive MS patients (Fig. 2c).

In addition to the serum cholesterol acceptance capacity, cellular ABC transporter expression contributes to the efflux capacity of monocytes. Therefore, we analyzed mRNA expression levels of the ABC transporters on monocytes of MS patients and controls. Whereas no changes in the expression of ABCA1 mRNA were observed between groups, the expression of ABCG1 was significantly (P < 0.05) lower on monocytes of RRMS patients compared to monocytes of healthy controls (Fig. 2d). Furthermore, in line with the increase in sHDL in low-BMI RRMS patients, we found a strong negative correlation between the amount of sHDL in the plasma and the capacity of serum to accept cholesterol via the ABCG1 transporter in RRMS patients with a low BMI (r = −0.94, P < 0.0001) (Fig. 2e). This correlation was not significant for control (r = −0.07, P = 0.67) or RRMS subjects (r = 0.04, P = 0.84) with a BMI higher than 23, nor for controls with a low BMI (r = −0.39, P = 0.09) (data not shown). These findings indicate that ABCG1-mediated cholesterol efflux capacity is reduced in RRMS patients, both at serum and at cellular level.

### HDL_3_ of low-BMI RRMS patients has a reduced capacity to suppress pro-inflammatory gene expression of human monocytes

**Figure 3:**
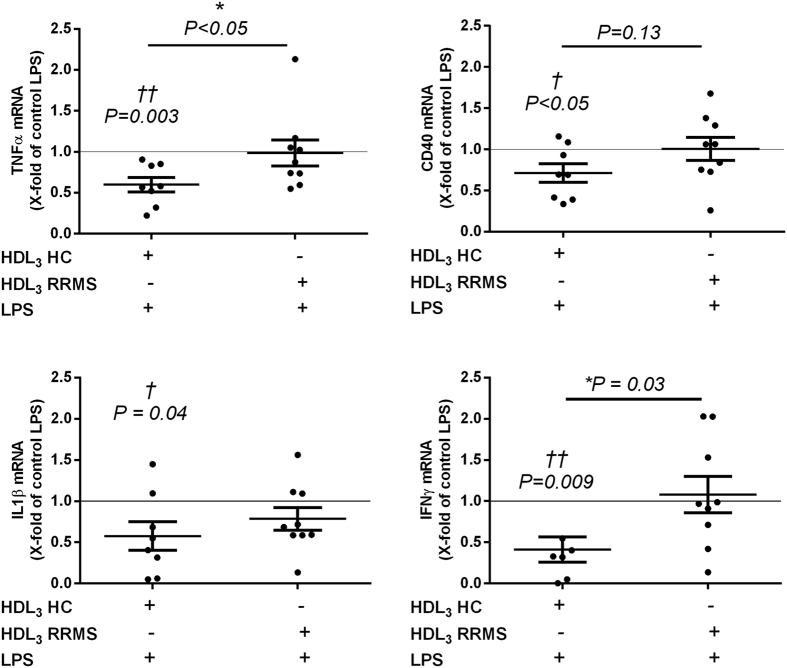
HDL_3_ of low-BMI RRMS patients does not suppress inflammation-induced gene expression of TNFα, CD40, IL1β, and IFNγ. Human monocytes from healthy donors were isolated from fresh blood samples. Monocytes of HC were pre-incubated with pooled HDL_3_ (60 mg/dl) isolated from low-BMI control subjects (n = 8) or low-BMI RRMS patients (n = 9) in low serum culture medium for four days followed by an overnight LPS (100 ng/ml) stimulus. Monocyte gene expression was measured using qPCR. Results are expressed as fold change of control LPS conditions without HDL_3_. HC = healthy controls; RRMS = relapsing-remitting multiple sclerosis; LPS = lipopolysaccharide. *P < 0.05, ^†^versus control LPS ( = 1) (^†^P < 0.05, ^††^P < 0.01).

Subsequently, we determined whether HDL_3_ from low-BMI RRMS patients retained its anti-inflammatory effects on human monocytes. To this end, we assessed the capacity of HDL_3_ to suppress the expression of pro-inflammatory genes that are involved in MS pathology by monocytes^46^,^47^. Our data show that, in contrast to sHDL from HC, sHDL from RRMS patients was not capable to suppress inflammation-induced pro-inflammatory gene expression of tumor necrosis factor alpha (TNFα), CD40, IL1β, and IFNγ in monocytes obtained from healthy controls (Fig. 3). These results demonstrate that the anti-inflammatory capacity of HDL_3_ is reduced in RRMS patients with a low BMI. Additionally, the reduced anti-inflammatory capacity of HDL_3_ of low-BMI RRMS patients was validated for TNFα protein levels (see: Supplemental Data Fig. 1). No effects of HDL_3_ of low-BMI HC or RRRMS patients were observed on monocyte expression levels of the anti-inflammatory genes CD163, CD206, and TGFβ (see: Supplemental Data Fig. 2).

### Tyrosine and tryptophan residues of ApoA-I in HDL_3_ of RRMS patients are modified

**Figure 4:**
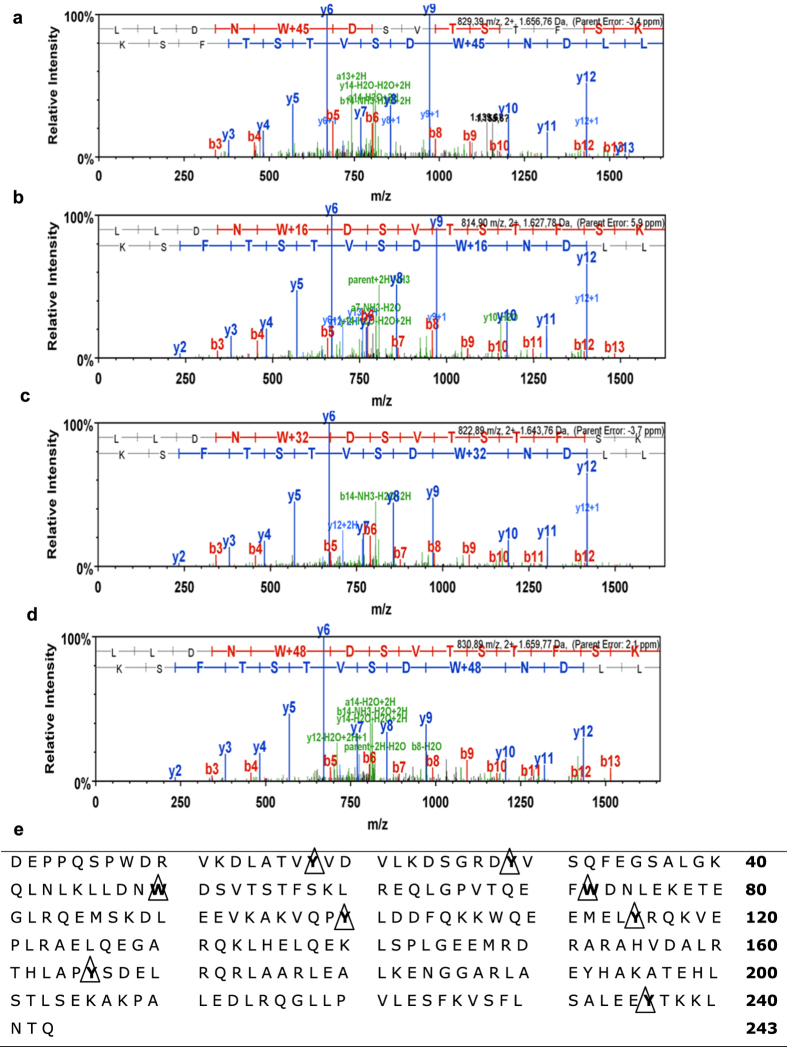
ApoA-I of RRMS patients is modified at its tyrosine and tryptophan residues. (**a–d**) Collision-induced dissociation (CID) spectra of Trp-50 nitration **(a)**, and Trp-50 mono-, di-, and tri-oxidation **(b,c** and **d** respectively). Spectra were acquired on analysis of in-gel tryptic digests of the ApoA-I band from HDL_3_ isolated with sequential flotation ultracentrifugation. Modifications were detected in an LC-MS/MS experiment as described under Methods. CID fragment ion annotation was according to the nomenclature by Roepstorff and colleagues^108^. (**e)** Summary of ApoA-I modification sites. Residues that were modified are indicated with ∆. The numbering of the amino acids cited refers to the amino acid sequence of the mature protein.

Inflammation-induced modifications of HDL proteins are shown to affect its function^12^,^22^,^48^,^49^. To identify possible protein changes on HDL of RRMS patients, ApoA-I tryptic peptides were analyzed for possible modifications with LC-MS/MS. ApoA-I of RRMS patients showed nitration on its Trp-50 residue (Fig. 4a). Furthermore, mono-, di-, and tri-oxidated peptides were detected on both Trp-50 (Fig. 4b,c and d respectively) and Trp-72 (see: Supplemental Data Fig. 3). In addition, we identified nitrations on Tyr-18, 29, 166, and 236 (see: Supplemental Data Fig. 4), and a modification on Tyr-18, 29, 100, 115, 166, and 236, corresponding to a mass increase of 126 atomic mass units (amu) (see: Supplemental Data Figure 5). An overview of the mentioned modification sites along the protein sequence of mature ApoA-I is shown in Fig. 4e. Altogether, our results show that HDL of RRMS patients is modified on its ApoA-I tyrosine and tryptophan residues, which may affect HDL function.

## Discussion

In this study, we determined whether lipoprotein levels and HDL function are altered in MS patients. Our data show that RRMS patients have smaller total LDL compared to healthy controls and to progressive MS patients, as a result of the reduced number of large LDL particles. The progressive MS patients are significantly older than the HC and RRMS patients. As we found that large LDL particle count increases with age, the difference in large LDL particle count between progressive and relapsing-remitting MS patients, but not between HC and RRMS patients, can be due to age. Smaller LDL particles are described to have an increased susceptibility to oxidation^50^,^51^ and a decreased LDL receptor affinity^52^ which can promote pro-inflammatory properties of LDL in RRMS patients. In a subgroup of RRMS patients with a low BMI (BMI ≤ 23 kg/m^2^), a higher level of sHDL, accompanied by increased levels of TG, large VLDL, and VLDL-TG, and a higher LP-IR was observed. This corresponds to the findings of Palavra and colleagues who observed an increase in sHDL and TG in the total RRMS patient population^41^. Interestingly, in their study population the average BMI of RRMS patients was lower (e.g. 23.75 kg/m^2^) compared to our study population (e.g. 25.9 kg/m^2^), which may explain why they observed an increase of sHDL in the total RRMS group and we only found this increase in the low-BMI group. Although the number of low BMI patients in the RRMS group was relatively low (n = 10), the finding that low BMI patients differ significantly from the other subjects was calculated based on the entire group of controls (n = 89) and patients (n = 36 RRMS, n = 25 progressive MS), and the statistical model predicting the effect observed in the group of RRMS patients was highly significant. It would be interesting to perform a larger study in which the focus lies on low-BMI RRMS patients in order to gain more insight into potential variables contributing to the altered lipoprotein profile observed in this specific subgroup of MS patients. LCAT mass, being highly correlated with its enzyme activity and its molar cholesterol esterification rate^45^, could not explain the observed increase in sHDL in low-BMI RRMS patients. Of note, cholesteryl ester transfer protein (CETP) activity can contribute to smaller HDL by removing CE from HDL, and should be addressed in future studies.

We found a reduced capacity of serum from RRMS patients to accept cholesterol via ABCG1, in combination with a reduced ABCG1 mRNA expression in monocytes. These data indicate that in addition to changes in the quantity of sHDL, HDL function is impaired in RRMS patients. Furthermore, these results suggest that the reduced capacity of serum in combination with the lower expression of ABCG1 on monocytes may result in an even more pronounced reduction in cholesterol efflux from MS monocytes *in vivo*. Cholesterol efflux is one of the main anti-inflammatory effects of HDL because it is responsible for maintaining cellular cholesterol homeostasis, and hereby regulates the cellular inflammatory phenotype^4^,^53^. In line with this, our data show that HDL_3_ of low-BMI RRMS patients is no longer effective in suppressing an inflammation-induced transcriptional profile of TNFα, CD40, IL1β and IFNγ in monocytes. We did not observe an effect of HDL on the expression of the anti-inflammatory markers CD206, CD163, and TGFβ. Although Sanson and colleagues reported an induction of several anti-inflammatory genes in murine macrophages^54^, other reports on the effect of HDL on human monocytes show no upregulation of anti-inflammatory genes after incubation with HDL, similar to our results^4^,^55^.

Since myeloid cells in the CNS of MS patients are largely derived from infiltrated monocytes^56^, the predetermined phenotypic and functional characteristics of these cells in the periphery can have important consequences for their contribution to MS pathology. Altogether, these findings indicate that the cholesterol acceptance and anti-inflammatory capacity of HDL are impaired in RRMS patients, which may contribute to disease progression.

An increase in IR, measured by the IR homeostasis model assessment (HOMA-IR) method, has been reported in MS patients, and was reported to be associated with invalidity scores (EDSS)^57^. In contrast to these findings, we found no association between LP-IR and EDSS scores (r = −0.109, P = 0.781) in the total RRMS patient group, nor in RRMS patients with a low BMI, making it unlikely that physical inactivity is responsible for the observed differences in our study. The anti-inflammatory MS-therapeutic interferon-β (IFN-β) can also induce IR by inhibiting insulin-induced tyrosine phosphorylation of the insulin receptor substrate 1 protein^58^. In this study, however, we found no effect of IFN-β therapy, neither of other patient therapies on the LP-IR or on other measured lipoprotein parameters. Although IR is most common in individuals with excess weight and excess fat around the waist^59^,^60^,^61^, IR in lean subjects is not uncommon^62^,^63^. Moreover, even in the absence of obesity, the administration of inflammatory cytokines in animals causes IR^64^. In addition, elevated levels of TNFα and IL-6, two cytokines that are involved in MS pathology^65^,^66^,^67^, are proposed to increase IR in patients with chronic inflammatory conditions^68^,^69^. This study did not investigate IR directly by means of a HOMA or oral glucose tolerance test. However, the NMR-derived LP-IR measurement is strongly associated with both the homeostasis model assessment of insulin resistance (r = 0.51) hereby being reflective of hepatic IR, and with glucose disposal rates (r = −0.53) hereby reflecting peripheral insulin sensitivity (85).

Besides an increase in IR, impaired glucose tolerance (IGT) and decreased insulin sensitivity are described in MS patients^31^,^70^. IR and IGT precede T2D and drive an accelerated risk for cardiovascular disease (CVD) by modulating the lipoprotein profile and influencing several mechanisms affecting the endothelium, the vascular wall, smooth muscle cells, and platelets^71^,^72^,^73^. Altogether, the shift towards sHDL together with smaller LDL, increased TG, larger and more TG-rich VLDL, and a higher LP-IR in the lipoprotein profile of low-BMI RRMS patients in this study are typical features of an insulin resistant, pre-T2D-like lipoprotein profile^43^,^44^. In T2D and CVD patients, not only a similar lipoprotein profile, but also impaired HDL-mediated cholesterol efflux and antioxidant function^74^,^75^,^76^,^77^, and a reduced expression of ABCG1 on monocytes is found, the latter which is described to lead to an increase in cellular cholesterol accumulation^78^. Together, these findings may suggest a pre-diabetic state in low-BMI RRMS patients. However, studies that determined the impact of co-morbidities like T2D and CVD on MS mortality rates are inconclusive^79^,^80^. Although the risk to develop CVD in the total MS population is similar to that in controls, the mortality rate due to CVD is higher in MS patients^81^, and vascular co-morbidities present during the disease course of MS can increase the risk of ambulatory disability^82^. Importantly, low-BMI MS patients have an increased mortality rate, which is highest in male subjects with cardiovascular disease as an underlying cause of death^83^. We found a negative correlation between small HDL and BMI in RRMS patients. However, this correlation was absent in progressive MS patients. This indicates that the observed changes in lipoprotein levels may be related to the ongoing inflammatory process which is predominant in RRMS patients compared to progressive patients^84^. Interestingly, pro-inflammatory monocyte subsets were found to be positively associated with levels of small HDL in atherosclerosis patients^85^. Pro-inflammatory monocyte subsets are suggested to contribute to the inflammatory responses in MS patients^86^, but their possible association with lipoprotein levels needs further clarification.

Changes in HDL function can be caused by modifications of the proteome and lipidome of HDL^87^,^88^,^89^. Using mass spectrometry, we identified the presence of nitrations and oxidations of tyrosine and tryptophan along with a + 126 amu modification on tyrosine residues in HDL_3_-derived ApoA-I of RRMS patients. Some of these changes in ApoA-I have been described to affect HDL function^48^. Interestingly, levels of the leukocyte-derived haemprotein myeloperoxidase (MPO), which is described to modify ApoA-I *in vitro*^90^ and *in vivo*^48^, are increased in MS patients^91^,^92^. Therefore, MPO may be involved in the observed ApoA-I modifications and impaired HDL function. In addition to these observations, we found a modification of + 126 amu, for which three possible modification candidates can be suggested: iodination, glycation (162 amu minus two water molecules) and octanoylation. Merely the latter modification is consistent with the observed significant increase in retention time of the modified peptide. The presence of these modifications on tyrosine and tryptophan indicates that these aromatic residues are preferred modification sites of ApoA-I. The exposition of the Trp domains in the center of the ApoA-I ring structure probably makes them more vulnerable for modifications. Importantly, the Trp-50 and Trp-72 domains are both responsible for lipid-binding of ApoA-I^93^, which is essential for small HDL to mature to larger HDL particles. Furthermore, interactions between the aromatic residues Tyr-100, 115 and 236 stabilize lipid-free ApoA-I particles^93^. Altogether, our results show that ApoA-I of RRMS patients is modified at its tyrosine and tryptophan residues, but the exact contribution of these modifications to HDL dysfunction in MS needs further investigation.

In summary, our data show that RRMS patients display an altered lipoprotein profile, which is especially present in RRMS patients with a low BMI. Furthermore, the anti-inflammatory and cholesterol acceptance properties of HDL are impaired, which may enhance the inflammatory response and promote disease progression in MS. In addition, the HDL alterations may predispose MS patients to additional health risks such as CVD and T2D. Further studies are needed to determine the impact of reduced HDL function on MS disease progression and co-morbidities. Knowledge on disturbances in lipoprotein levels and function may help to explain the variability in MS disease course and outcome, and may pave the way for the development of tailored therapies for specific MS patient subgroups.

## Materials and Methods

### Subjects

A total of 89 controls, 36 RRMS, and 25 progressive MS patients were recruited after providing their informed consent. Relapsing-remitting patients were in remission when participating in the study, as at time of sampling there was no evidence of an active flare up. Progressive MS patients include both secondary progressive MS patients and progressive relapsing MS patients. MS patients were included independent of their medication status. An overview of the therapies in the patient subgroups is shown in Supplemental Data Table 1. Exclusion criteria for patients and controls were reported hypercholesterolemia, cardiovascular diseases, diabetes, pregnancy, cancer, liver disease, and treatment with cholesterol modifying agents. The mean time since diagnosis for all RRMS patients was 8.8 ± 1.1 years. The mean time since diagnosis for low-BMI RRMS patients was 13 ± 3.4 years.

### Sample processing

EDTA-plasma was obtained after centrifugation of fasting blood samples at 400 g for ten minutes and another ten minutes at 1,500 g to remove remaining cells. Cells were cultured freshly. Plasma was immediately stored at −80 °C by the University Biobank Limburg (UBiLim). All plasma samples were analyzed within 6 months after collection. The NMR analysis was performed for 89 controls, 36 RRMS and 25 progressive MS patients. For the other experiments the maximum amount of samples available in the University Biobank were used for each experiment. Study information on healthy controls (age, gender, weight, and length) and MS patients (age, gender, weight, length, therapy, EDSS score, time since disease onset, and relapse rate) was obtained from UBiLim. The mean EDSS for the RRMS patients was 2.3 ± 0.3 (for low-BMI RRMS patients 3.2 ± 0.6), and for the progressive MS patients 4.9 ± 0.3. The study was approved by the Medical Ethics Committees of Hasselt University and UZ Leuven. All procedures followed were in accordance with institutional guidelines and regulations.

### Nuclear magnetic resonance spectroscopy

Fasting plasma samples were thawed and immediately analyzed using the 400-MHz proton Vantera Clinical Analyzer^®^ (Liposcience, Raleigh, North Carolina)^94^, the first NMR providing lipoprotein tests approved by the US Food and Drug Administration for use as a clinical instrument source. Particle size, concentration and subclass (large, medium, small) count were measured for all lipoprotein classes (HDL, LDL, VLDL). In addition, TG concentrations and VLDL-TG were measured. The NMR analysis involved measurement of the sample, and the deconvolution of the signal and conversion of the signal into specific lipoprotein subclass concentrations. The 400-MHz proton NMR spectrum of each plasma sample was measured, producing a signal at ~0.8 ppm, which was derived from the methyl group protons of the lipids carried in the lipoprotein subclasses. The signals at ~0.8 ppm had unique and distinctive frequencies and line shapes, each of which were accounted for in the deconvolution analysis model. The lipoprotein insulin resistance (LP-IR) index measured by the NMR is calculated from three lipoprotein subclasses (large VLDL, small LDL and large HDL) and three particle sizes (VLDL, LDL and HDL). Although the LP-IR is an indirect measure of IR, it is strongly associated with both the homeostasis model assessment of insulin resistance (r = 0.51), hereby being reflective of hepatic IR, and with glucose disposal rates (r = −0.53) hereby reflecting peripheral insulin sensitivity^95^. The results are reported on a scale ranging from 0 (most insulin sensitive) to 100 (most insulin resistant).

### LCAT mass

Lecithin - cholesterol acyltransferase (LCAT) mass was measured with a commercial ELISA (Alpco Diagnostics, Salem, New Hampshire)^96^. In brief, an anti-LCAT monoclonal antibody (MoAb) coated 96-well plate was incubated for 2 hours with freshly thawed fasting plasma samples and standards in duplicate. Unbound material was washed away, and horseradish peroxidase-labeled anti-LCAT MoAb was added for 1 hour. After the incubation and subsequent washes, the antibody/LCAT/enzyme complex was incubated with a substrate solution for 15 minutes and terminated with a stop reagent for another 15 minutes. The color intensity of the enzyme reaction was measured with a microplate reader at 492 nm.

### ABCA1 and ABCG1 cholesterol efflux capacity

Serum was derived from plasma by adding calcium^97^. ATP-binding cassette transporter A1 (ABCA1) - and ABCG1 -specific cholesterol efflux to serum was quantified using baby hamster kidney (BHK) cells expressing the mifepristone-inducible human ABCA1 or ABCG1 as described previously^98^,^99^. In brief, mock and transfected BHK cells expressing the mifepristone inducible human ABCA1 cDNA were cultured in 10% FCS (Atlanta Biologicals, Norcross, Georgia) labeled for 18 hours with 1 μCi/ml 3H-cholesterol (Perkin Elmer, Waltham, Massachusetts) in DMEM (Life technologies, Grand Island, New York) supplemented with 0.01% BSA and 10 nM Mifeprestone (Life technologies). Excess cholesterol was washed away with 1x phosphate buffered saline (PBS). DMEM medium containing 0.01% BSA and 1% of the serum of controls or MS patients was added as a cholesterol acceptor together with 10 nM Mifeprestone to induce ABC transporter specific cholesterol efflux for 4 h. After 4 hours, media were collected and filtered through a 1.2 μm 96-well filter plate (Pall Corporation Life Sciences, Port Washington, New York) and cells were lysed in 0.5 ml of 0.1% SDS and 0.1 M NaOH. Liquid scintillation counting (MicroBeta 1450 Liquid Scintillation, Perkin Elmer) was used to measure radioactive counts of cholesterol in media and cell fractions. Results were expressed as the percentage of total counts effluxed into the media after 4 hours of efflux. HDL is known to be the major lipoprotein contributing to the process of cholesterol efflux, but the use of whole serum for this experiment is more physiologically relevant than using solely HDL.

### Monocyte isolation and cell culture

After separation from plasma, blood was diluted with 1x PBS without Ca2 + and Mg2 + (Lonza, Verviers, Belgium) (ratio 1:1), supplemented with 2 mM EDTA. The diluted blood samples were subjected to density gradient separation on Ficoll-Paque (ratio 2:1) (GE Healthcare Life Sciences, Buckinghamshire, UK) and centrifuged for 20 minutes at 400 g. After centrifugation the layer of peripheral blood mononuclear cells (PBMCs) was collected and washed twice in 1x PBS supplemented with 2 mM EDTA. CD14 + monocytes were isolated from PBMCs using EasySep™ human CD14 positive selection (Stemcell Technologies, Grenoble, France) according to manufacturer’s instructions. After isolation, cells were re-suspended in RPMI 1640 supplemented with 0.5% penicillin/ streptomycin, 1% non-essential amino acids, 1% sodium pyruvate and 2.5% FCS (low serum culture medium) and placed in 24 well culture plates at a density of 0.5 × 106 cells per well. Cells were pre-incubated with HDL_3_ (60 mg/dl) or left untreated for four days, followed by an overnight stimulus with 100 ng/ml lipopolysaccharide (LPS) (E. coli O55:B5, Sigma-Aldrich, Diegem, Belgium). Previous dose response studies within our group and studies by others have shown that this LPS concentration gives a good induction of pro-inflammatory genes in human monocytes^100^,^101^.

### Isolation of HDL_3_ from plasma

Butylated hydroxytoluene (BHT, Sigma-Aldrich) was added (50 μg/ml) to freshly isolated plasma immediately to protect lipoproteins from auto-oxidation during the isolation procedure. HDL_3_ (density 1.125–1.210 g/ml) were isolated using sequential density floatation ultracentrifugation^102^. After isolation, HDL_3_ was dialyzed extensively at 4 °C against 1x PBS and afterwards against low serum culture medium for use in cell cultures. Lipopolysaccharide (LPS) content in isolated HDL_3_ was determined using the Chromogenic Limulus Amebocyte Lysate assay kit (Genscript Incorperation, Aachen, Germany). Isolated HDL_3_ contained a neglectable amount of endotoxins (<1.8 × 10^−4^ pg/μg HDL_3_). HDL_3_ were sterilized using a 0.22 μM filter, stored in sealed tubes under a layer of nitrogen at 4 °C in the dark to prevent auto-oxidation, and were used within two weeks after isolation.

### Real-time quantitative reverse transcription polymerase chain reaction

Total RNA was isolated using the High Pure RNA isolation kit (Qiagen, Venlo, the Netherlands) according to manufacturer instructions. Total RNA concentration and RNA purity were verified with a Nanodrop Spectrophotometer ND-1000 (Isogen Life Science, St-Pieters-Leeuw, Belgium). RNA was reverse transcribed using a cDNA synthesis kit (Quanta, Gaithersburg, USA). Gene expression was measured with quantitative PCR (qPCR) using the StepOnePlus™ Real-Time PCR System (Applied Biosystems, Halle, Belgium). Fast SYBR Green (Applied Biosystems), 0.3 μM forward and reverse primers and 12.5 ng cDNA were used per qPCR reaction using universal cycling conditions (10 ’ 95 °C, 15 ” 95 °C and 1’ 60 °C, 40 cycles)^103^. PCR products were loaded on 2% agarose gels to confirm the specificity of amplification and the absence of primer dimer formation. Data were analyzed using the ΔΔCt method^104^ and are shown as fold change over controls. Expression levels were normalized using the most stable housekeeping genes, determined with geNorm^105^. The used primer sequences for real time PCR are listed in Supplemental Data Table 2.

### Mass spectrometry

ApoA-I SDS PAGE and in-gel digestion – The protein concentration of HDL_3_ was determined using the bicinchoninic acid assay (Thermo Scientific, Erembodegem, Belgium), with albumin as the standard. HDL_3_ (3 μg) isolated by sequential ultracentrifugation was separated by Tris-glycine SDS-PAGE on 12% polyacrylamide gels for 1 hour at 200 V. The Coomassie Brilliant Blue G-250 stained ApoA-I band with an apparent Mr of 23 kDA was cut from the gel, destained and digested overnight at 37 °C using sequencing-grade porcine trypsin (Promega, Leiden, the Netherlands). The tryptic peptides extracted from the gel, were speedvac dried and stored at −20 °C until analysis. LC-MS/MS analysis – An Easy-nLC1000 liquid chromatograph (Thermo Scientific, Erembodegem, Belgium) was on-line coupled to a mass calibrated LTQ-Orbitrap Velos Pro (Thermo Scientific) via a Nanospray Flex ion source (Thermo Scientific) using sleeved 30 μm ID stainless steel emitters (spray voltage + 2.3 kV, capillary temperature: 200 °C)^106^. The SpeedVac dried tryptic peptide mixture was dissolved in 40 μl buffer A (0.1% v/v formic acid in Milli-Q water) of which 10 μl was loaded, concentrated and desalted on a trapping pre-column (Acclaim PepMap 100 C18, 75 μm ID × 2 cm nanoViper, 3 μm, 100 Å, Thermo Scientific) at a buffer A flow rate of 5 μl/min for 5 minutes. The peptide mixture was separated on an Biosphere C18 column (50 μm ID × 20 cm, 5 μm, 120 Å, NanoSeparations, Nieuwkoop, the Netherlands) at a flow rate of 100 nL/min with a linear gradient in 180 minutes of 0 to 70% buffer B (0.1% v/v formic acid in acetonitrile) in buffer A. MS data were acquired in a data-dependent mode under direct control of the Xcalibur software v 2.2, selecting the fragmentation events based on the top six precursor abundances in the survey scan (350–2000 Th). Data analysis - The mass spectrometric RAW data was analyzed using Proteome Discoverer software v.1.4 (Thermo Scientific) with build-in Sequest v.1.4 and interfaced with an in-house Mascot v.2.5 server (Matrix Science, London, UK). MS/MS spectra were searched against the SwissProt human fasta database (Version June 2015; 42121 entries) and peptide scoring for identification was based on following search criteria: enzyme trypsin, maximum missed cleavages 2, precursor mass tolerance 30 ppm and fragment mass tolerance 0.5 Da. Carbamidomethylation of cysteine and oxidation of methionine, histidine and tryptophan were set as fixed and dynamic modifications, respectively. Error tolerant searches in Mascot were used to detect unspecified peptide modifications using the Unimod database collection of protein modifications for mass spectrometry^107^. Output files of both search engines were uploaded and automatically validated in Scaffold v.4.4 (Proteome Software) using the Peptide Prophet and Protein Prophet algorithm with a preset minimal peptide and protein identification probability of 95% and 99%, respectively. The peptide false discovery rate was < 0.5*%*.

### Statistical analysis

For descriptive statistics, one way ANOVA or Kruskal – Wallis tests were performed in GraphPad Prism (Windows version 6). Differences in gender were assessed using chi-square tests. Univariate multiple linear regression models for each dependent response variable were fitted using SAS (Windows version 9.4). For response variables for which the error normality assumption was not met, logarithmic or square root transformations were performed. A backward-elimination approach was performed in each univariate multiple linear regression model (until all remaining variables had P < 0.05), starting from the model “response = group age BMI gender group*BMI group*age group*gender BMI*age BMI*gender age*gender”. A post hoc Tukey or Bonferroni test was used to correct for multiple testing. Statistical significance is presented as *P < 0.05, **P < 0.01, ***P < 0.001, and ****P < 0.0001 in all figures. The Pearson correlation coefficient was used for linear regression analysis with continuous variables. The absence of multicollinearity in the linear regression models was verified by calculating the variance inflation factor. Continuous variables are presented as mean ± SEM. The authors had full access to and take full responsibility for the integrity of the data.

## Additional Information

**How to cite this article:** Jorissen, W. *et al*. Relapsing-remitting multiple sclerosis patients display an altered lipoprotein profile with dysfunctional HDL. *Sci. Rep.* **7**, 43410; doi: 10.1038/srep43410 (2017).

**Publisher's note:** Springer Nature remains neutral with regard to jurisdictional claims in published maps and institutional affiliations.

## Supplementary Material

Supplementary Information

## Figures and Tables

**Table 1:** Descriptive statistics for the study population.

	HC	RRMS	Prog MS
**N**	89	36	25
**Age**	42.0 ± 1.5	42.0 ± 1.7	51.7 ± 1.7^**, ††^
**Male gender, %**	38 (43%)	10 (28%)	11 (44%)
**BMI**	24.9 ± 0.4	25.9 ± 0.7	24.5 ± 0.9
**Conventional lipid panel**			
Total cholesterol, mg/dl	182.7 ± 3.8	169.6 ± 5.3	187.4 ± 7.9
Triglycerides, mg/dl	113.6 ± 6.1	136.6 ± 17.8	106.2 ± 5.6
HDL-c, mg/dl	61.0 ± 1.7	59.3 ± 2.3	60.8 ± 3.5
LDL-c, mg/dl	109.8 ± 3.8	96.6 ± 4.8	115.6 ± 6.3
**Lipoprotein subclasses**			
**HDL**			
Size nm	9.5 ± 0.1	9.4 ± 0.1	9.5 ± 0.1
Total particle count, μmol/L	35.8 ± 0.6	36.3 ± 0.9	34.2 ± 1.1
Small particle count, μmol/L	14.9 ± 0.9	15.2 ± 1.4	15.7 ± 1.2
Medium particle count, μmol/L	12.1 ± 0.8	12.8 ± 1.3	9.0 ± 1.3
Large particle count, μmol/L	7.5 ± 0.4	7.1 ± 0.5	8.1 ± 0.7
**LDL**			
Size, nm	21.0 ± 0.1	20.6 ± 0.1^*, ‡^	21.0 ± 0.1
Total particle count, nmol/L	1, 182 ± 44.9	1, 099 ± 60.4	1, 217 ± 67.2
Small particle count, nmol/L	463.9 ± 34.8	517.0 ± 62.7	418.2 ± 69.5
Large particle count, nmol/L	432.9 ± 24.3	312.1 ± 39.8^*, ‡‡^	503.7 ± 51.4
**IDL**			
Particle count, nmol/L	166.7 ± 11.9	157.1 ± 18.1	170.6 ± 24.8
**VLDL**			
Size, nm	51.5 ± 0.8	53.6 ± 1.6	50.78 ± 1.2
Total particle count, nmol/L	43.9 ± 2.8	48.9 ± 4.4	41.2 ± 3.5
Small particle count, nmol/L	22.3 ± 1.5	24.9 ± 2.4	22.9 ± 2.5
Medium particle count, nmol/L	17.6 ± 1.8	17.6 ± 2.2	15.3 ± 1.7
Large particle count, nmol/L	5.0 ± 0.5	7.3 ± 1.7	4.2 ± 0.7
VLDL-Triglycerides, mg/dl	78.7 ± 5.2	100.6 ± 15.3	70.8 ± 5.2
**LP-IR index** (0–100)	45.9 ± 2.5	50.9 ± 3.5	41.2 ± 3.8

Values are means ± SEM. HC = healthy controls; RRMS = relapsing-remitting MS; Prog MS = progressive MS. * versus controls (*P < 0.05, **P < 0.01); ^††^ versus RRMS (^††^P < 0.01), ^‡‡^versus progressive MS (^‡‡^P < 0.01).

**Table 2:** Fitted univariate multiple regression models for the study population.

Response Cholesterol Parameter	CONFOUNDING PARAMETERS for the Study Population			
	Age	Gender	Group	Group*BMI
**LDL-z (nm)**		F > M^†^ ***	RRMS < HC^‡^ * and RRMS < Prog MS^‡^ *	
**Large LDL-p (nmol/L)**	↑ with age ^§^ y = 167.3 + 4.5x	F > M^||^ **	RRMS < HC ^#^ ** and RRMS < Prog MS^‡^ *	
**Small HDL-p (μmol/L)**		M > F^&^ ****		RRMS > HC^‡^ with a BMI ≤ 23*

^†^for individuals of the same group; ^‡^ of the same gender; ^§^ of the same group and gender, ^||^ of the same group and age; ^#^ of the same age and gender; ^&^of the same group and BMI. - z = particle size; - p = particle count; M = male; F = female. Responses are shown only when the covariate “Group” was among the confounders. The interactions Age*Gender, Age*BMI, Age*Group and BMI*Gender were not confounding for any of the shown responses. *P < 0.05, **P < 0.01, ***P < 0.001, and ****P < 0.0001.

**Table 3:** Insulin resistant-like risk factors in low-BMI RRMS patients.

Response Cholesterol Parameter	Controls (n = 29)	RRMS (n = 10)	Prog MS (n = 11)
**LP-IR (0–100)**	40.98 ± 3.47	67.50 ± 9.10^*, ††^	31.47 ± 6.75
**TG (mg/dl)**	100.86 ± 7.98	264.44 ± 20.73 ^****, ††††^	99.22 ± 15.37
**VLDL-TG (mg/dl)**	71.30 ± 6.47	197.98 ± 17.00^****, ††††^	58.88 ± 12.60
**VLDL-z (nm)**	51.09 ± 1.52	63.49 ± 3.99^*, †^	49.05 ± 2.96
**HDL-c (mg/dl)**	63.59 ± 3.02	59.09 ± 5.28	67.88 ± 4.9
**LDL-c (mg/dl)**	99.68 ± 6.32	104.2 ± 10.76	126.73 ± 10.26

Values are means ± SEM. -z = particle size; -c = cholesterol; *versus controls (*P < 0.05, ****P < 0.0001); ^†^versus Prog MS (^†^P < 0.05, ^††^P < 0.01, ^††††^P < 0.0001).
