# Supplementary material for: Relapsing-remitting multiple sclerosis patients display an altered lipoprotein profile with dysfunctional HDL
Source: Sci Rep. 2017 Feb 23;7:43410. doi: 10.1038/srep43410 (PMC5322497; doi:10.1038/srep43410)
Supplement: Supplementary Information [file srep43410-s1.pdf]

SUPPLEMENTARY INFO FILE

Relapsing-remitting multiple sclerosis patients display an altered lipoprotein profile with dysfunctional HDL

Winde Jorissen [1]; Elien Wouters [1]; Jeroen F. Bogie [1]; Tim Vanmierlo [1]; Jean-Paul Noben [1]; Denis Sviridov [2]; Niels Hellings [1]; Veerle Somers [1]; Roland Valcke [3]; Bart Vanwijmeersch [4]; Piet Stinissen [1]; Monique T. Mulder [5]; Alan T. Remaley [2]; and Jerome J.A. Hendriks [1*]

[1] Hasselt University, Dept. of Immunology and Biochemistry, Biomed, Diepenbeek, Belgium

(**Postal address:** Agoralaan, Building C, 3690 Diepenbeek, Belgium; **e-mail address:** winde.jorissen@uhasselt.be, elien.wouters@uhasselt.be, jeroen.bogie@uhasselt.be, tim.vanmierlo@uhasselt.be, jeanpaul.noben@uhasselt.be, niels.hellings@uhasselt.be, veerle.somers@uhasselt.be, piet.stinissen@uhasselt.be, Jerome.hendriks@uhasselt.be)

[2] NIH, Dept. of Laboratory Medicine, Clinical Center, Bethesda, United States

(**Postal address**: Bldg. 10/Rm. 2C-433, 10 Center Drive, Bethesda, MD. 20892, United States; **e-mail address:** sviridovd@nhlbi.nih.gov, aremaley1@nhlbi.nih.gov)

[3] Hasselt University, Faculty of Sciences, Molecular and Physical Plant Physiology, Diepenbeek, Belgium

(**Postal address:** Agoralaan, Building D, 3690 Diepenbeek, Belgium; **e-mail address:** roland.valcke@uhasselt.be)

[4] Revalidation and MS Center, Overpelt, Belgium

(**Postal address**: Boemerangstraat 2, 3900 Overpelt, Belgium; **e-mail address:** bart.vanwijmeersch@uhasselt.be)

[5] Erasmus MC, Dept. of Vasc. and Met. diseases, Rotterdam, the Netherlands

(**Postal address:** 's-Gravendijkwal 230, 3015 CE Rotterdam, the Netherlands; **e-mail address:** m.t.mulder@erasmusmc.nl)

*Corresponding author:

Jerome J.A. Hendriks, Hasselt University (BIOMED), Agoralaan Building C, 3590 Diepenbeek, Belgium

email: jerome.hendriks@uhasselt.be

Tel: +32 (0) 11 26 92 07 Fax: +32 (0) 11 26 92 99

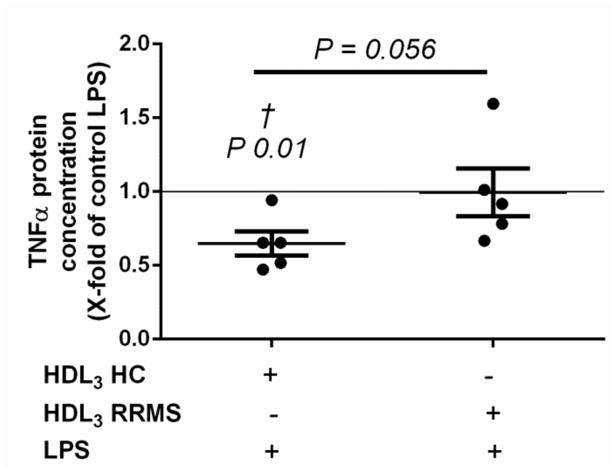

Supplemental Fig. 1. HDL_3 of low-BMI RRMS patients does not suppress inflammation-induced protein expression of TNFα Monocytes of HC were pre-incubated with pooled HDL_3 (60 mg/dl) isolated from low-BMI control subjects (n=8) or low-BMI RRMS patients (n=9) for four days followed by an overnight LPS (100 ng/ml) stimulus. Protein expression levels of TNFα present in the medium of monocytes were measured with ELISA. Results are expressed as fold change of control LPS conditions without HDL_3. HC = healthy controls; RRMS = relapsing-remitting multiple sclerosis; LPS = lipopolysaccharide. † versus control LPS (=1) (†$P<0.05$).

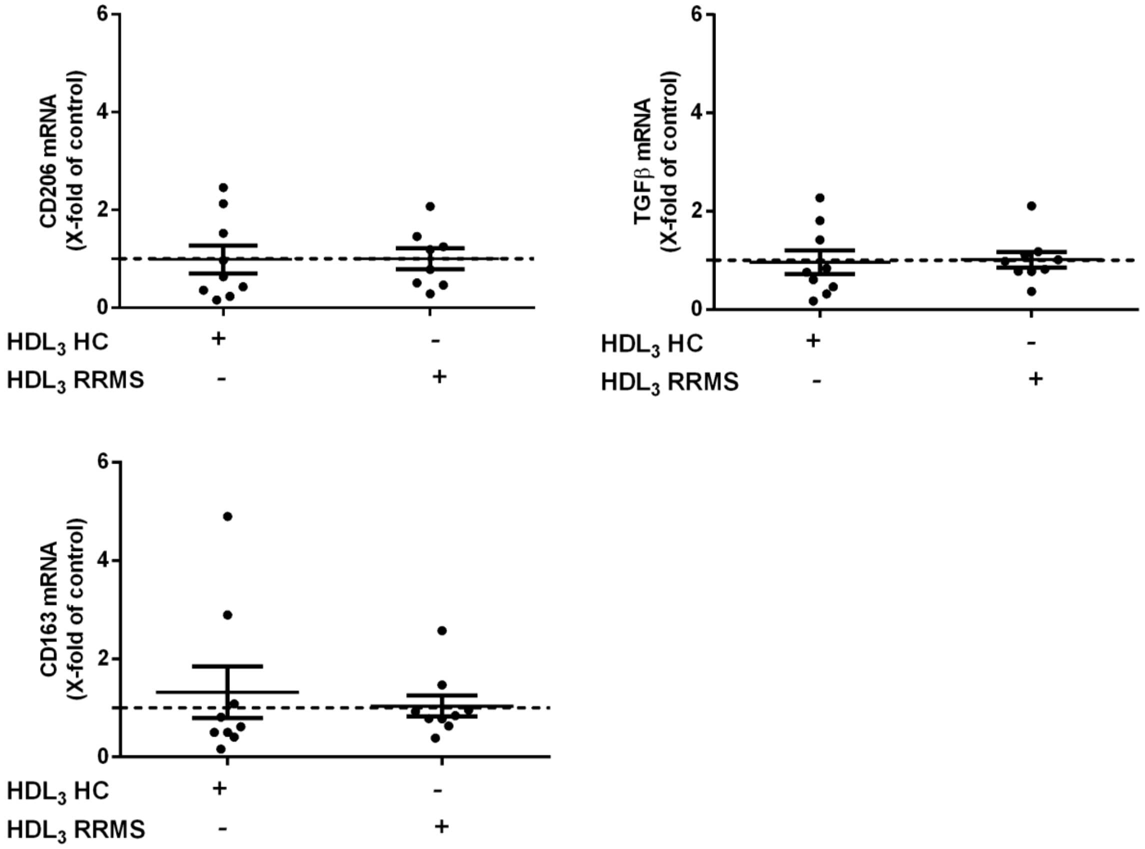

Supplemental Fig. 2. HDL_3 of low-BMI HC and RRMS patients does not influence gene expression of the anti-inflammatory markers CD163, TGFβ, and CD206 on monocytes Monocytes of HC were pre-incubated with pooled HDL_3 (60 mg/dl) isolated from low-BMI control subjects (n=8) or low-BMI RRMS patients (n=9) for four days. mRNA expression levels of CD163, TGFβ, and CD206 were measured with qPCR. Results are expressed as fold change of control conditions without HDL_3. HC = healthy controls; RRMS = relapsing-remitting multiple sclerosis.

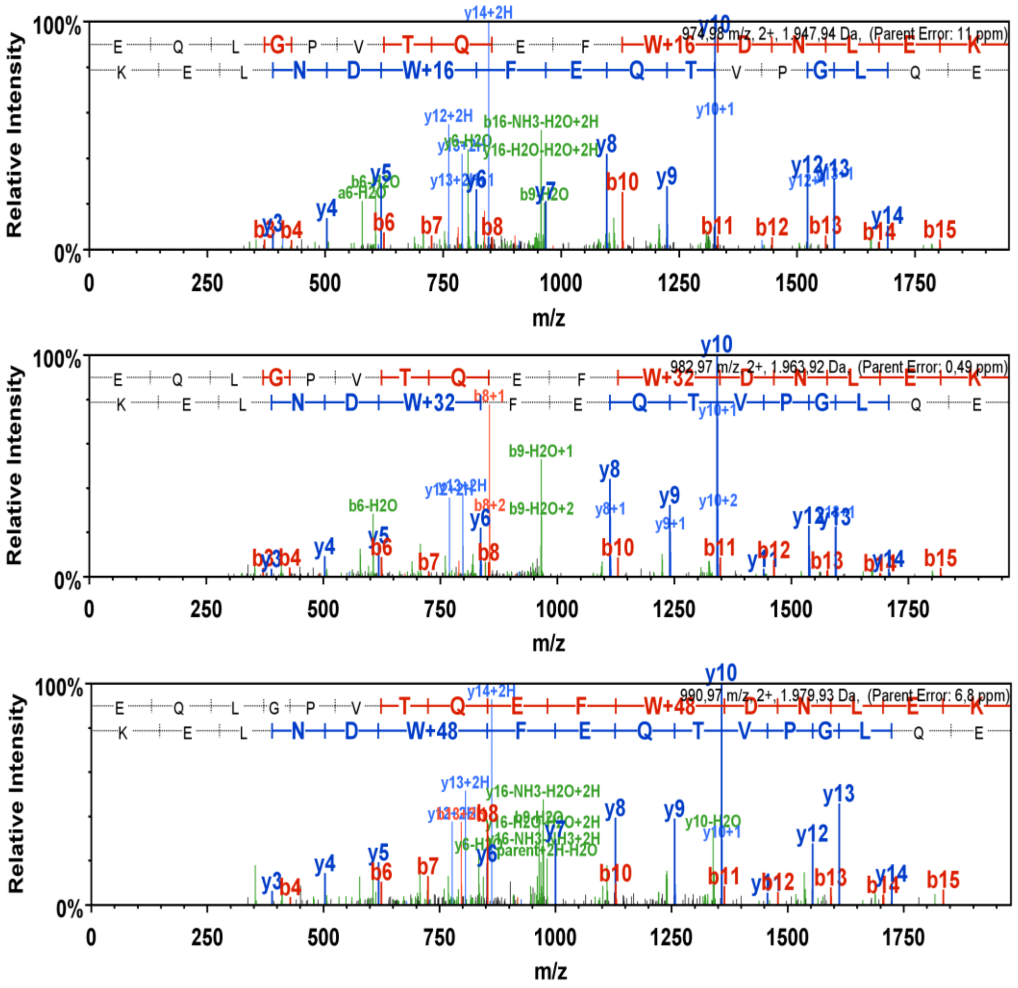

Supplemental Fig. 3. Mono-, di- and tri-oxidations of Trp-72 in ApoA-I of RRMS patients CID spectra of mono-, di- and tri-oxidations of Trp-72 in ApoA-I of RRMS patients are shown respectively from top to bottom. Spectra were acquired during the analysis of in-gel tryptic digests of the ApoA-I band from sHDL isolated with sequential flotation ultracentrifugation. Modifications were detected in an LC-MS/MS experiment as described under “Methods”.

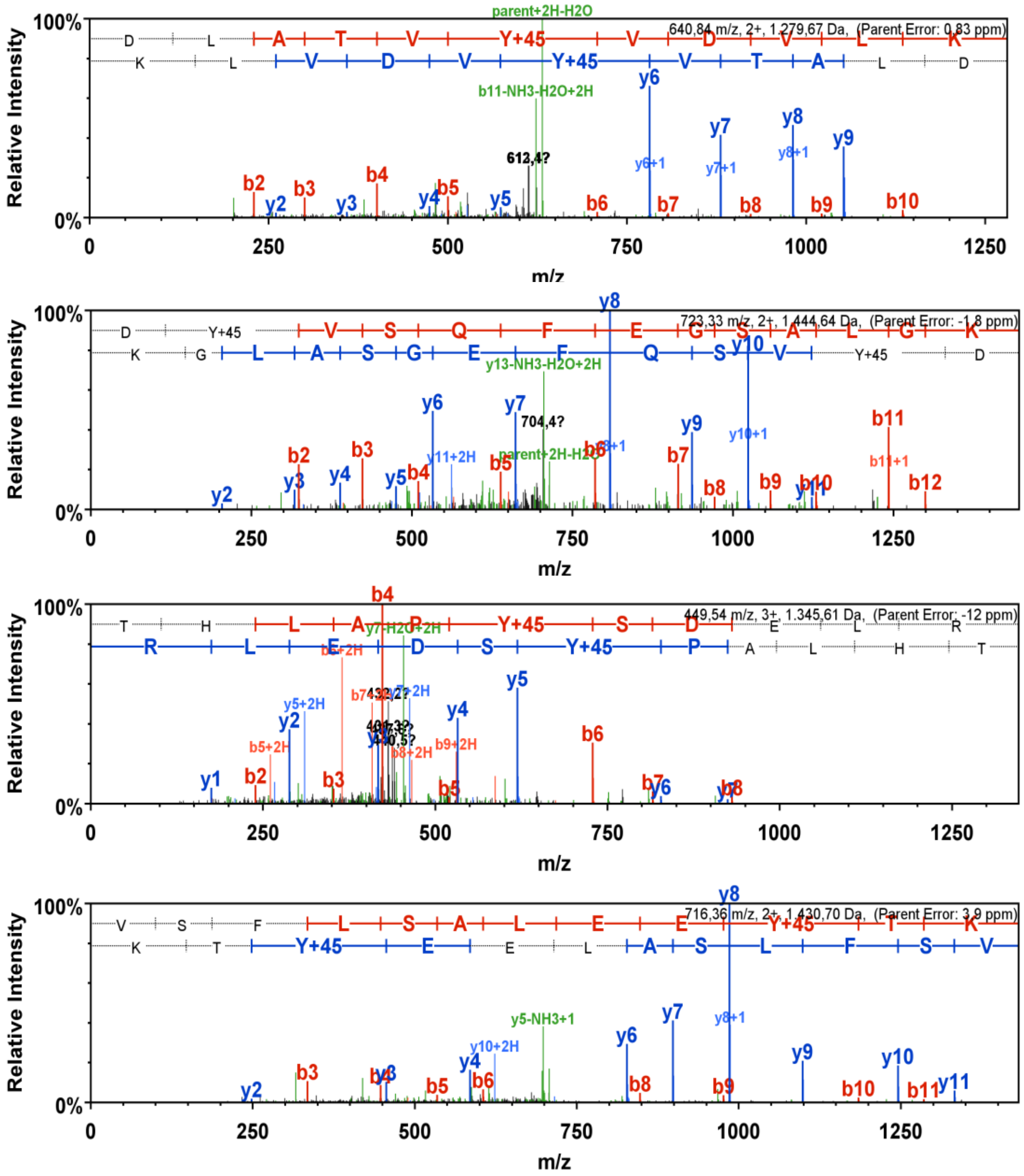

Supplemental Fig. 4. Nitrations of Tyr-18, 29, 166, and 236 in ApoA-I of RRMS Patients CID spectra of Tyr-18, 29, 166, and 236 nitrosation sites in ApoA-I of RRMS patients are shown respectively from top to bottom.

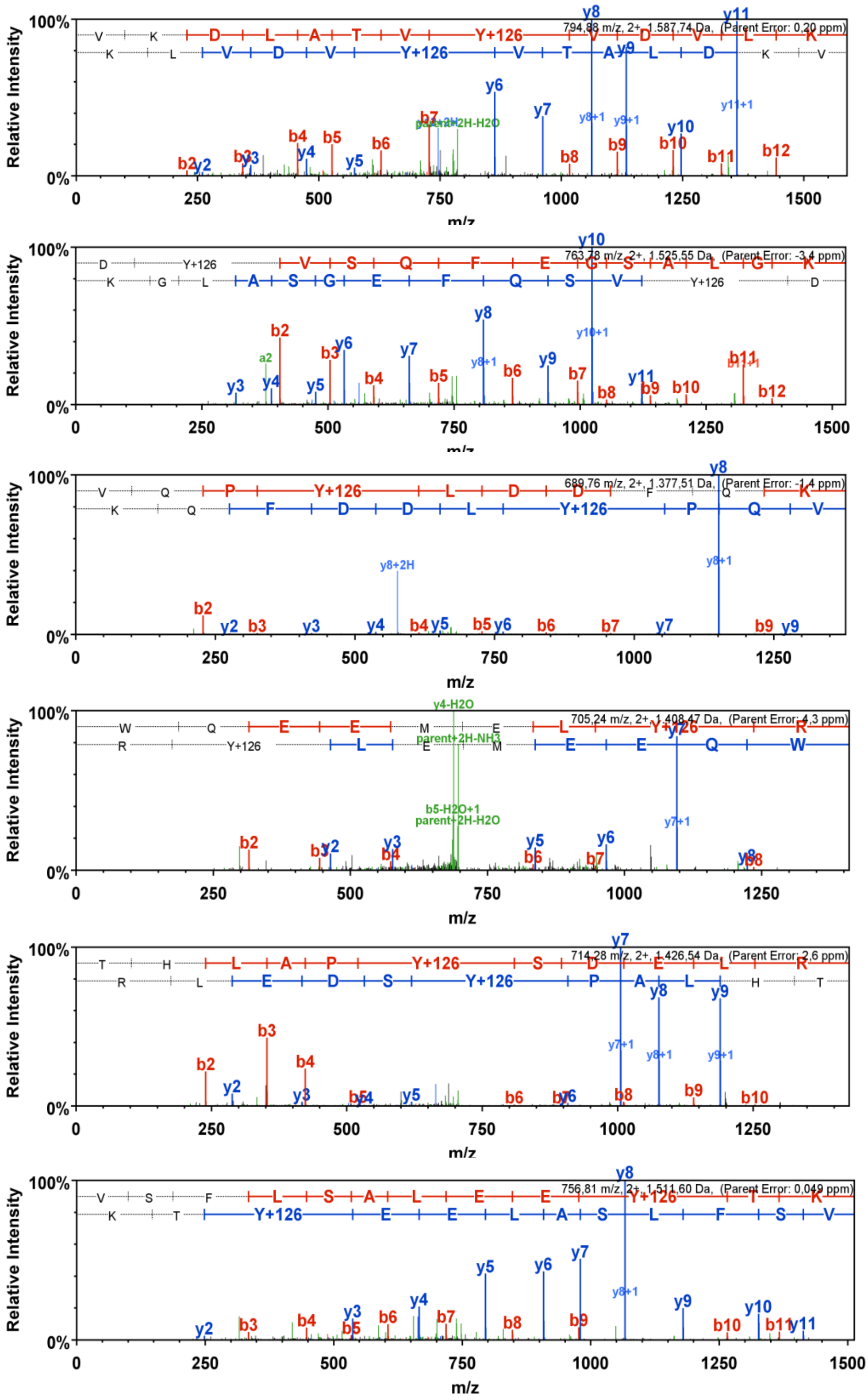

Supplemental Fig. 5. 126 amu modification of Tyr-18, 29, 100, 115, 166, and 236 in ApoA-I of RRMS Patients CID spectra of 126 amu modifcations of Tyr-18, 29, 100, 115, 166, and 236 in ApoA-I of RRMS patients are shown respectively from top to bottom.

Supplemental Table 1. MS patient therapies

	RRMS (n)	Progressive MS (n)
No treatment	5	9
Interferon β (Rebif®)	10	1
Glatiramer acetate (Copaxone®)	2	2
Cyclofosfamide (Endoxan®)	3	10
Fingolimod (Gilenya®)	9	1
Natalizumab (Tysabri®)	5	1
Teriflunomide (Aubagio®)	1	
Methotrexate (Ledertrexate®)		1
Dimethyl Fumarate (BG-12®)	1	

Supplemental Table 2. Primer sequences for qPCR

	**Forward**	**Reverse**
Human TNFα	AGCCCATGTTGTAGCAAACC	TGAGGTACAGGCCCTCTGAT
Human CD40	TGCGACCCCAACCTAGGGCTT	AAAGCCGGGCGAGCATGAGC
Human IL1β	GATGAAGTGCTCCTTCCAGG	GCATCTTCCTCAGCTTGTCC
Human IFNγ	GGGGCCAACTAGGCAGCCAAC	AAGCACTGGCTCAGATTGCAGGC
Human CD206	TCTCCTACTGGACACCAGGC	ATTTCTGTGATTCGGCATCC
Human CD163	TGGAGTGACCTGCTCAGATG	CATCACACACTGTTCCCCAC
Human TGFβ	GTGGAAACCCACAACGAAAT	CACGTGCTGCTCCACTTTTA
Human ABCA1	AACGAGACTAACGAGGCAATC	ACACAATACCAGCCCAGAAC
Human ABCG1	CCAGAAGTCGGAGGCCATC	AAGTCCAGGTACAGCTTGGCA
